# Recent exploration of γ-AApeptide based antimicrobial peptide mimics as potential therapeutics towards drug-resistant bacteria

**DOI:** 10.37349/eds.2025.100888

**Published:** 2025-02-19

**Authors:** Jarais Fontaine, Jianfeng Cai

**Affiliations:** Department of Chemistry, University of South Florida, Tampa, FL 33620, USA

**Keywords:** Antimicrobial peptides, peptidomimetics, γ-AApeptides, amphipathic

## Abstract

Over the last 60 years, only four new classes of antibiotics have been introduced, while the prevalence of antibiotic-resistant Gram-positive and Gram-negative bacteria has risen. This underscores the urgent need for new antibacterial therapeutics. This commentary leverages the recent exploration of γ-substituted-*N*-acylated-*N*-aminoethyl amino acid peptides (γ-AApeptides) to mimic the structures and function of natural antimicrobial peptides (AMPs), highlighting the promise and limitations for developing a new, effective treatment for antibiotic-resistant bacteria.

## Introduction

In 1939, gramicidin A as a peptide was discovered from the soil bacterium *Bacillus brevis*, marking the first commercially available antibiotic and a cornerstone for the development of antimicrobial therapeutics [1]. According to the Database of Antimicrobial Activity and Structure of Peptides (DAASP), over 22,000 peptides with antimicrobial properties have been identified to date [2]. Antimicrobial peptides (AMPs) are evolutionary biomolecules formed as part of the defense mechanisms of numerous organisms [3]. Despite their prevalence and diversity, AMPs typically contain fewer than 50 amino acids with positive charges ranging from +2 to +11, enabling interaction with negatively charged bacterial membranes through electrostatic interactions and hydrophobic residues for membrane disruption and penetration. AMPs could be characterized by their secondary structures, predominantly α-helix or β-sheet [4]. Through careful manipulation of hydrophobic and hydrophilic residues, AMPs exhibit broad-spectrum antimicrobial activity and immunomodulatory properties with negligible bacterial resistance and low toxicity [5]. However, AMPs face several limitations, including high manufacturing costs, high in vivo toxicity, low in vivo efficacy, susceptibility to proteolytic degradation, and rapid clearance [6].

**Figure 1.**
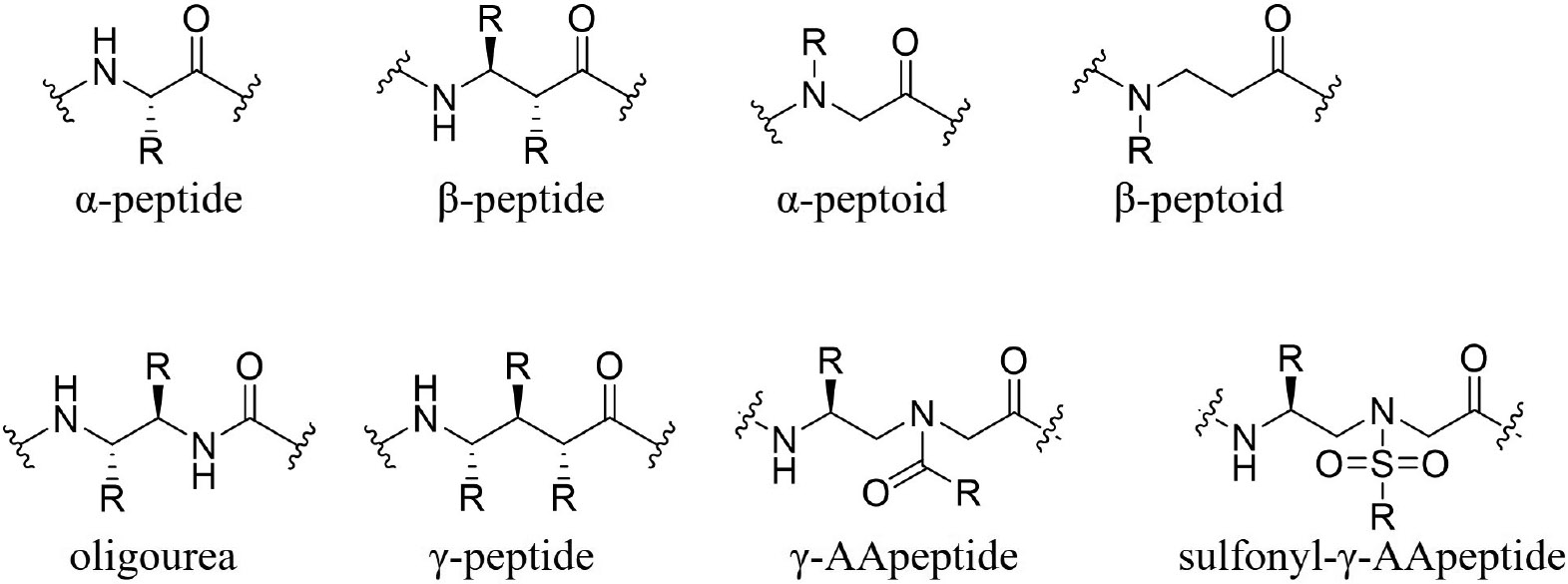
Structures of different peptidomimetic backbone scaffolds. γ-AApeptide: γ-substituted-*N*-acylated-*N*-aminoethyl amino acid peptide

Researchers are exploring various strategies to overcome these challenges. For instance, Nazeer et al. [7] reported the stabilization of macrocyclic AMPs through cross-linked swapping, enhancing conformation rigidity and antimicrobial activity via side chain-to-side chain lactam cross-links. Other approaches include transdermal delivery methods, such as incorporating nanoparticles to encapsulate AMPs, allowing for controlled release and resistance to enzymatic degradation [6]. The limitations of naturally occurring AMPs have driven the development of synthetic AMP mimetics, including small molecule AMP mimics, cyclic peptides (including stapled peptides), and peptidomimetics like γ-substituted-*N*-acylated-*N*-aminoethyl amino acid peptides (γ-AApeptides) developed by our lab (Figure 1). Among these, sulfonyl-γ-AApeptides are notable for forming α-helical secondary structures and exhibiting enhanced proteolytic degradation stability and cell permeability [8]. It could be a viable strategy to employ sulfonyl-γ-AApeptides to address the barriers surrounding AMPs and meet the growing need for therapeutics against drug-resistant bacteria. Alternatively, there have been several successful reported cases of synthetic peptidomimetics with potent antimicrobial activity, for example, Zhang et al. [9] demonstrated the eradication of *Staphylococcus aureus* from the incorporation of β-peptides. In 2022, Tallet et al. [10] reported a series of oligourea inspired peptides consisting of strategically distributed histidine arginine, tryptophan, and γ-valine residues to mimic the amphiphilic nature of AMPs. Very recently Firdous et al. [11] developed a series of short lipidated urea-containing α/β hybrid peptides which showed excellent antibacterial activity against both Gram-positive and Gram-negative bacteria.

## Peptidomimetics as synthetic AMPs

**Figure 2.**
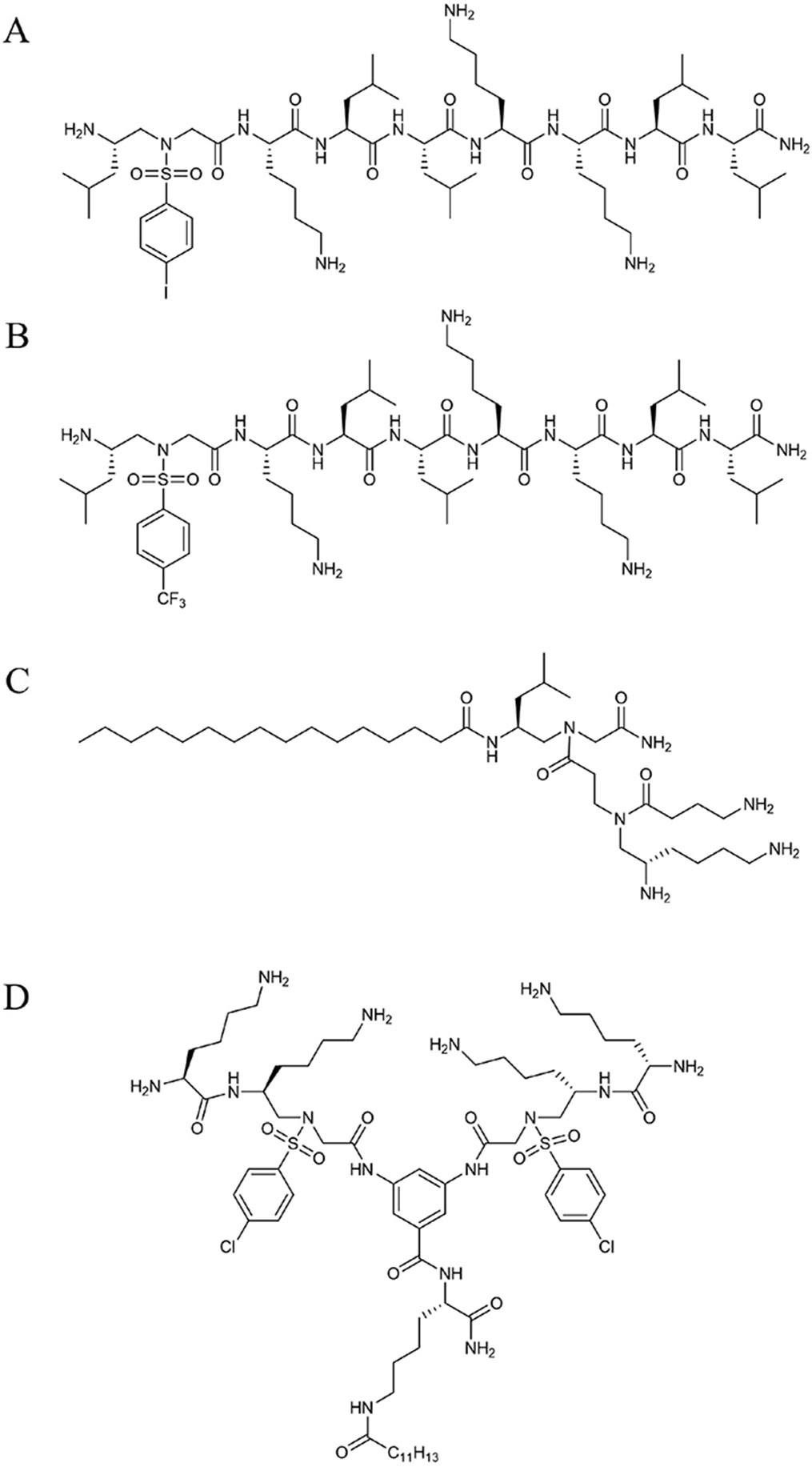
Structures of discussed antimicrobial peptides. (**A**) Structure of K122; (**B**) structure of CF_3_-K11; (**C**) structure of YW-11; (**D**) structure of compound 17

Derived from feleucin-bombinin-1 (BO1), the cationic nonapeptide feleucin-K3 exhibits an amphiphilic α-helical structure with potent antimicrobial activity but fails to meet clinical therapeutic requirements due to low selectivity and poor stability. To this end, sulfonyl-γ-AApeptide building blocks were introduced at either the C or N terminus of the sequence with varying hydrophobic side chains to develop analogs. It was discovered that substituting the first two hydrophobic side chains of K3 with a sulfonyl-γ-AApeptide containing more hydrophobic side chains resulted in more potent antimicrobial activity, particularly against Gram-positive bacteria, K122 (Figure 2A). The addition of a sulfonyl-γ-AA unit led to negligible hemolytic activity, enhanced stability against mouse serum, and improved selectivity compared to K3 [12].

Within the world of medicinal chemistry, it is well known that by incorporating fluorine or fluorinated groups, one could potentially overcome protease degradation as well as substantially improve pharmacokinetic properties through its modulation of pKa influencing the bioavailability of a compound [13]. To this extent, authors theorized that by introducing fluorinated groups to sulfonyl-γ-AApeptide, a further enhanced feleucin-K3 inhibitor was developed. As seen in the structure of CF_3_-K11, it bears vast similarities to K122; however, it contains a fluorocarbon residue in the para position of a benzene ring (Figure 2B). This resulting influence of such a modification leads to the facilitation of hydrogen bonding stabilizing the secondary structure, as well as enhancing salt stability while maintaining its potent antibacterial activity [14].

A subclass of γ-AApeptides explored recently as potential antibacterial agents are short lipidated dendrimeric γ-AApeptides. We hypothesized that introducing a positively charged γ-AA building block to a lipidated hydrophobic γ-AA building block could mimic the amphiphilic nature of canonical AMPs. It was found that building blocks conjugated with C16 tails exhibited effective antimicrobial activity, particularly YW-1, which demonstrated the ability to disrupt cell membranes with good selectivity and low hemolytic toxicity (Figure 2C) [15]. The addition of a long hydrophobic tail on γ-AApeptides, a method our group first employed in 2012, showed unprecedented potential as a novel antibiotic therapeutic due to its large diversification potential, ease of synthesis, stability in the presence of serum, and potent selective activity against fungi and clinically relevant Gram-positive and Gram-negative bacteria [16]. Since then, functionalized γ-AApeptides were investigated to mimic not only the antimicrobial aspects of AMPs but also the immunomodulatory response, as seen in the cyclic γ-AApeptides we reported in 2014 [17].

To take advantage of the amphiphilic nature of lipidated sulfonyl-AApeptides, we examined the influence of dimerization to interact more strongly with the negatively charged surface of bacterial membranes. We synthesized a series of multiple dimeric lipo-α/sulfonyl-γ-AA hybrid peptides consisting of varying hydrophobic tail lengths, cationic groups, and hydrophobic achiral side chains to assess their influence on mimicking AMPs. From this series, we noted lead compound 17 possessed potent antibacterial activity with high selectivity towards bacteria cells. Similar to previously reported sulfonyl-γ-AA containing peptides, compound 17 exhibited excellent stability with seemingly no degradation after 24 h in the presence of serum (Figure 2D). The bacterial resistance was tested against a common antibiotic, ciprofloxacin, and revealed compound 17’s reduced susceptibility towards bacterial resistance. These promising results allow for further exploration of dimeric lipo-α/Sulfonyl-γ-AA hybrid peptides as potential AMP mimics [18].

## Future directions

Despite significant advances in the development of AMP mimics, there have been no new FDA-approved antibiotic treatments. Our continued efforts to address the challenges associated with AMPs, such as toxicity, pharmacokinetic profiles, and low in vivo activity against drug-resistant Gram-negative bacteria, through the functionalization of γ-AApeptides are promising. However, our lab has greater success in targeting Gram-positive bacteria, such as methicillin-resistant *Staphylococcus aureus* (MRSA) and methicillin-resistant *Staphylococcus epidermis* (MRSE), likely due to the ability of amphiphilic peptidomimetics to penetrate and disrupt the thick peptidoglycan layer of Gram-positive bacteria. In contrast, Gram-negative bacteria, with their thin peptidoglycan layer protected by an outer membrane, present a more challenging target. It is our hope that through the fine-tuning of hydrophobic and hydrophilic residues, we can design peptidomimetics with potent broad-spectrum antibacterial activity as potential therapeutics. Looking forward, our efforts are also geared towards developing AMPs targeting specific bacteria such as *Clostridium difficile*. Overall, we hope to inspire future researchers to aid in the development of AMP mimetics as potential therapeutics towards the fight against drug-resistant bacteria.

## Abbreviations

AMPs: antimicrobial peptides
γ-AApeptides: γ-substituted-*N*-acylated-*N*-aminoethyl amino acid peptides

## References

[1] Takada Y, Itoh H, Paudel A, Panthee S, Hamamoto H, Sekimizu K, Discovery of gramicidin A analogues with altered activities by multidimensional screening of a one-bead-one-compound library. Nat Commun. 2020;11:4935. 33004797 10.1038/s41467-020-18711-2 PMC7531004

[2] Pirtskhalava M, Amstrong AA, Grigolava M, Chubinidze M, Alimbarashvili E, Vishnepolsky B, DBAASP v3: database of antimicrobial/cytotoxic activity and structure of peptides as a resource for development of new therapeutics. Nucleic Acids Res. 2021;49:D288–97. 33151284 10.1093/nar/gkaa991 PMC7778994

[3] Ebenhan T, Gheysens O, Kruger HG, Zeevaart JR, Sathekge MM. Antimicrobial peptides: their role as infection-selective tracers for molecular imaging. Biomed Res Int. 2014;2014:867381. 25243191 10.1155/2014/867381 PMC4163393

[4] Mahlapuu M, Håkansson J, Ringstad L, Björn C. Antimicrobial Peptides: An Emerging Category of Therapeutic Agents. Front Cell Infect Microbiol. 2016;6:194. 28083516 10.3389/fcimb.2016.00194 PMC5186781

[5] Teng P, Shao H, Huang B, Xie J, Cui S, Wang K, Small Molecular Mimetics of Antimicrobial Peptides as a Promising Therapy To Combat Bacterial Resistance. J Med Chem. 2023;66:2211–34. 36739538 10.1021/acs.jmedchem.2c00757

[6] Firdous SO, Sagor MMH, Arafat MT. Advances in Transdermal Delivery of Antimicrobial Peptides for Wound Management: Biomaterial-Based Approaches and Future Perspectives. ACS Appl Bio Mater. 2024;7:4923–43. 10.1021/acsabm.3c00731 37976446

[7] Nazeer N, Kooner N, Ghimire A, Rainey JK, Lubell WD, Meneksedag-Erol D, Secondary Structure Stabilization of Macrocyclic Antimicrobial Peptides via Cross-Link Swapping. J Med Chem. 2024;67:8693–707. 38771638 10.1021/acs.jmedchem.4c00005

[8] Sang P, Shi Y, Huang B, Xue S, Odom T, Cai J. Sulfono-γ-AApeptides as Helical Mimetics: Crystal Structures and Applications. Acc Chem Res. 2020;53:2425–42. 32940995 10.1021/acs.accounts.0c00482 PMC8903043

[9] Zhang K, Du Y, Si Z, Liu Y, Turvey ME, Raju C, Enantiomeric glycosylated cationic block co-beta-peptides eradicate *Staphylococcus aureus* biofilms and antibiotic-tolerant persisters. Nat Commun. 2019;10:4792. 31636263 10.1038/s41467-019-12702-8 PMC6803644

[10] Tallet L, Frisch E, Bornerie M, Medemblik C, Frisch B, Lavalle P, Design of Oligourea-Based Foldamers with Antibacterial and Antifungal Activities. Molecules. 2022;27:1749. 35268850 10.3390/molecules27051749 PMC8911826

[11] Firdous S, Sarkar AR, Manhas R, Chowdhary R, Rathore A, Kumari J, Synthesis, Characterization, and Antimicrobial Activity of Urea-Containing α/β Hybrid Peptides against *Pseudomonas aeruginosa* and Methicillin-Resistant *Staphylococcus aureus*. ACS Omega. 2025;10:2102–15. 39866621 10.1021/acsomega.4c08680 PMC11755142

[12] Guo X, Yan T, Rao J, An Y, Yue X, Miao X, Novel Feleucin-K3-Derived Peptides Modified with Sulfono-γ-AA Building Blocks Targeting *Pseudomonas aeruginosa* and Methicillin-Resistant *Staphylococcus aureus* Infections. J Med Chem. 2023;66:1254–72. 36350686 10.1021/acs.jmedchem.2c01396

[13] Purser S, Moore PR, Swallow S, Gouverneur V. Fluorine in medicinal chemistry. Chem Soc Rev. 2008;37:320–30. 18197348 10.1039/b610213c

[14] Guo X, Miao X, An Y, Yan T, Jia Y, Deng B, Novel antimicrobial peptides modified with fluorinated sulfono-γ-AA having high stability and targeting multidrug-resistant bacteria infections. Eur J Med Chem. 2024;264:116001. 38056301 10.1016/j.ejmech.2023.116001

[15] Wang Y, Xue M, Gao R, Chakraborty S, Wang S, Zhao X, Short, Lipidated Dendrimeric γ-AApeptides as New Antimicrobial Peptidomimetics. Int J Mol Sci. 2023;24:6407. 37047380 10.3390/ijms24076407 PMC10094648

[16] Niu Y, Padhee S, Wu H, Bai G, Qiao Q, Hu Y, Lipo-γ-AApeptides as a new class of potent and broad-spectrum antimicrobial agents. J Med Chem. 2012;55:4003–9. 22475244 10.1021/jm300274p

[17] Padhee S, Smith C, Wu H, Li Y, Manoj N, Qiao Q, The development of antimicrobial α-AApeptides that suppress proinflammatory immune responses. Chembiochem. 2014;15:688–94. 24677440 10.1002/cbic.201300709 PMC4043931

[18] Wei L, Gao R, Wang M, Wang Y, Shi Y, Gu M, Dimeric lipo-α/sulfono-γ-AA hybrid peptides as broad-spectrum antibiotic agents. Biomater Sci. 2021;9:3410–24. 33949388 10.1039/d0bm01955k PMC8903075

